# Effect of Dietary Crude Protein on Productive Efficiency, Nutrient Digestibility, Blood Metabolites and Gastrointestinal Immune Markers in Light Lambs

**DOI:** 10.3390/ani10020328

**Published:** 2020-02-19

**Authors:** Jonathan Pelegrin-Valls, Beatriz Serrano-Pérez, Daniel Villalba, María José Martín-Alonso, Juan Ramón Bertolín, Margalida Joy, Javier Álvarez-Rodríguez

**Affiliations:** 1 Departamento de Ciencia Animal, Universidad de Lleida, Av. Rovira Roure 191, 25198 Lleida, Spain; bserrano@ca.udl.cat (B.S.-P.); dvillalba@ca.udl.cat (D.V.); maralomj@gmail.com (M.J.M.-A.); jalvarez@ca.udl.cat (J.Á.-R.); 2 Centro de Investigación y Tecnología Agroalimentaria de Aragón, Av. Montañana 930, 50059 Zaragoza, Spain; jrbertolin@cita-aragon.es (J.R.B.); mjoy@cita-aragon.es (M.J.)

**Keywords:** dietary protein, feed intake, growth performance, urea, creatinine, oxidative status, gene expression

## Abstract

**Simple Summary:**

Animal production is considered to compete with human food due to land use for feed ingredients and their relationship with environmental pollution. Crude protein (CP) is vital for the normal development of growth and carcass conformation in lambs. Dietary CP may be dependent on breed, age and market weights at slaughter. However, it appears that the feed industry formulates dietary CP levels above the physiological needs of these animals. Therefore, this study was designed to assess the effect of reducing the level of dietary CP (−2%) in intensively-fed growing (14–19 kg of body-weight) and finishing (19–25 kg of body-weight) lambs. Our findings revealed that a 2% reduction of dietary protein did not negatively affect some selected nutrient metabolism or mucosal immune function markers in the gastrointestinal tract. In addition, it was observed that the growth performance and feed efficiency of lambs fed lower CP levels would be the same as those fed higher levels.

**Abstract:**

This study hypothesized that reducing the level of crude protein (CP) in lambs′ feed may improve nutrient utilization and did not negatively affect their productive efficiency, blood metabolites, oxidative status (OS) or intestinal immune barrier function. A total of 120 weaned male Ripollesa lambs (45–60 days old and 15.0 ± 1.5 kg of body weight) were used. Four feed concentrates were formulated for two different phases (growing and finishing): CP20/19 group (20% and 19% of CP on dry matter basis, for each phase, respectively) and CP18/17 group (18% and 17% of CP on dry matter basis, for each phase, respectively). Lambs were randomly assigned to feeding treatments by balancing initial body weight between groups. The reduction of dietary CP level did not impair their growth performance parameters, while it did improve the apparent digestibility of organic matter. Furthermore, the lambs of the CP18/17 group showed lower plasma urea levels with no effect on OS (malondialdehyde levels) or gastrointestinal immunity markers (gene expression of interleukin 10 (*IL10*), tumor necrosis factor-α (*TNFA*) and transforming growth factor-β1 (*TGFB*)).

## 1. Introduction

Spain is the second largest producer of sheep meat in Europe, after the United Kingdom [1]. In most Spanish farms, finishing lambs are raised until 20–25 kg of body weight (BW) by means of small framed breeds and intensive feeding systems based on concentrate and straw ad libitum. Dietary crude protein (CP) requirements are normally attained by including vegetable protein concentrates, such as soybean and rapeseed meal in feed, but a CP excess may increase the feed costs and the excreted nitrogen to the environment. Optimizing the use of feed ingredients in the lamb diet could improve the production efficiency, reduce feed costs and contribute to the mitigation of environmental emissions of ammonia (NH_3_) and nitrous oxide (N_2_O) [2,3], which may improve the fertilizer value of manure.

Currently, the Spanish feed industry formulates lamb concentrates containing between 15% and 21% of CP on a dry matter basis (DM) [4]. In general, the recommended dietary CP for local Spanish sheep breeds ranges from 19 to 21% of CP on DM basis in starter feed (up to 14–16 kg BW) and 16% to 20% of CP on DM basis in growing-finishing diets, depending on the growth potential of the breed (autochthonous or improved crossbreds) and the energy density of the feed [5]. In contrast, in early-maturing improved breeds, dietary CP requirements on a DM basis would range from 11.7% to 12.8% during finishing until heavy weights [6]. More recent studies have estimated that the dietary CP requirements would range from 16.2% to 15.1% on a DM basis in growing-finishing lambs between 15 and 25 kg of BW, respectively [7].

In addition, it is necessary to understand how the reduction of dietary CP affects the balance between the immune defense and tolerance mechanisms of the gastrointestinal tract. Low protein diets have been associated with states of oxidative stress that form free radicals and promote pro-inflammatory cytokines [8]. Consequently, the intestinal mucosa may be affected and develop an immune imbalance. In this case, anti-inflammatory cytokines would act as a tolerance mechanism [9]. An animal suffering from oxidative stress develops high levels of malondialdehyde (MDA) as a product of lipid peroxidation, which can be assessed in blood plasma as an indicator of oxidative status (OS) [10].

This study evaluated the effects of the reduction of dietary CP in lambs on the productive efficiency, selected blood metabolites associated with protein (urea and creatinine) and oxidative (MDA) status and apparent nutrient digestibility. Furthermore, the OS and the expression of pro-inflammatory and regulatory cytokines in ruminal and ileal tissue were assessed.

## 2. Materials and Methods

The animals were handled and slaughtered in accordance with the Spanish Animal Protection Regulations RD 53/2013, which complies with European Union Directive 2010/63 with regard to the protection of animals used for experimental and other scientific purposes. The lambs were raised in commercial conditions following the Council Directive 98/58/EC concerning the protection of animals kept for farming purposes.

### 2.1. Animals, Diets and Experimental Design

The experiment was conducted between January and May 2018 in the experimental facilities Nial of BonÀrea Agrupa, located in Guissona (Lleida, Catalonia, Spain, 41°46′32.2″ N, 1°16′33.2″ E; 484 m above sea level), where the lambs were kept in loose-housed sheds. The average monthly temperature for January, February, April and May was 5.3, 3.0, 11.7 and 14.4 °C, respectively.

A total of 120 weaned male breed Ripollesa lambs of 45–60 days old and 15.0 ± 1.5 kg of BW were used. The study was replicated in two equal batches of 60 lambs each: batch 1, from January to February (6 weeks) and batch 2, from April to May (6 weeks). The lambs of both batches were housed in 12 pens (5 animals/pen; 1.04 m^2^ per animal) and were distributed in homogeneous groups according to their initial BW.

The lambs were submitted to a two-phase feeding program: growing (14 to 19 kg BW), which lasted 21 days in both batches, and finishing (19 to 25 kg BW), which lasted 21 ± 2 days in batch 1 and 18 ± 5 days in batch 2. Four diets with different levels of CP were formulated and supplied in two treatments: the CP20/19 group (n = 60), which was fed with 20% and 19% of CP on DM basis in the growing and finishing phase, respectively; and the CP18/17 group (n = 60), which was fed with 18% and 17% of CP on DM basis in the growing and finishing phase, respectively. Each pen was randomly assigned to one of the diets, taking into account that each treatment had the same initial BW and had half of the pens with small (14.0 ± 1.2 kg BW) and large (16.0 ± 1.1 kg BW) framed animals. 

All diets were isoenergetic (1 UFC/kg) and they were formulated with the same ingredients and additives in the same manufacturing lot. Only the percentage of inclusion of vegetable protein concentrates was modified (Table 1). The growing phase diet included coccidiostatic (decoquinate). The feed presentation was granulated with a pellet diameter of 3.5 mm and the granulation temperature was 60 °C.

In the growing and finishing phase, in all pens, ad libitum water and cereal straw was offered, the nutritional value of which was: 90.5% of DM, 4.3% ash, 3.6% CP, 68.3% neutral-detergent fiber (NDF), 41.1% acid-detergent fiber (ADF) and 0.07% phosphorus (P).

### 2.2. Feed Intakes and Growth Performance

The offered concentrate and straw were recorded daily, and the refused quantities of both ingredients were recorded once weekly on a pen basis (6 replicates/group × 2 batches). Lambs were individually weighed once a week to calculate the average daily gain (ADG, g/day). The feed conversion rate (FCR, g/g) was calculated as the ratio between average daily concentrate intake and ADG. On the slaughter day, the lambs had water ad libitum and were fasted for 18–20 h. Pre-slaughter BW and carcass weight (kg) of half the lambs in each group (6 replicates/group × 2 batches) were recorded to calculate the carcass yield (%).

### 2.3. Blood Samples

Blood samples were collected in vacuum tubes with EDTA (5 mL) (BD Vacutainer^®^, Becton, Dickinson and Company, Plymouth, UK) from the jugular vein of 2 randomly selected animals per pen (6 replicates/group × 2 batches). The samples were taken at 8:00 a.m. in the last week of each feeding phase. The same lambs were sampled during the growing and finishing phases. After extraction, the samples were centrifuged in situ (3000× *g* for 10 min) to obtain the plasma and stored at −20 °C until analysis.

Plasma concentrations of urea (mg/dL) and creatinine (mg/dL) were determined as indicators of protein metabolism. Both metabolites were determined with an automatic analyzer (GernonStar, RAL/TRANSASIA, Dabhel, India). Reagents were provided by the analyzer manufacturer. For urea quantification, the kinetic method based on the enzyme urease was used to catalyze the hydrolysis of urea into ammonia and carbon dioxide. The test had a measurement range between 2 and 350 mg/dL. The mean intra- and inter-assay coefficients of variation of the test were 2.8% and 2.7%, respectively. The creatinine as final by-product of the muscular metabolism, that originates from the creatine, was quantified by means of the enzymatic method. The creatinine measurement range was 0.03 to 50 mg/dL. The mean intra- and inter-assay coefficients of variation of the test were 3.1% and 5.1%, respectively.

Plasma samples in lambs were treated to determinate the total MDA (TMDA) as a result of the quantification of free MDA (FMDA) and protein-bound (PBMDA) separately following the procedure of Yonny et al. [11]. Proteins of plasma were precipitated with trichloroacetic acid and separated from the supernatant by centrifugation. The FMDA was determined in the supernatant while the PBMDA was determined in the pellet, in both cases after the reaction of this MDA with 2-thiobarbituric acid (TBA) in acid medium (with trichloroacetic acid) and high temperatures (≈100 °C) to form the adduct MDA-TBA_2_ as indicated in Yonny et al. [11]. After this sample treatment, plasma concentrations of MDA as an oxidative biomarker (µM/L) was determined by liquid chromatography using an ACQUITY UPLC H-Class liquid chromatograph (Waters, Milford, Massachusetts, USA) equipped with a silica-based bonded phase column (Acquity UPLC HSS PFP, 100 mm × 2.1 mm × 1.8 µm, Waters), an absorbance detector (Acquity UPLC Photodiode Array PDA eλ detector, Waters) and a fluorescence detector (2475 Multi λ Fluorescence Detector, Waters). The quantification of MDA was by fluorescence detection at ʎ_excitation_ = 530 nm and ʎ_emission_ = 550 nm [12]. To quantify the MDA, an external linear curve calibration between 0.02 and 40 µM was used. More details of the chromatographic conditions used are described in Bertolin et al. [12].

### 2.4. Feces, Concentrate and Straw Samples

Feces samples (approximately 50 g) were collected at 8:00 a.m. by rectal stimulation at the end of each phase (growing and finishing) in at least 3 lambs from each pen to make a pool of feces (6 replicates/group × 2 batches). Concentrate samples, straw and feces were collected to estimate the apparent nutrient digestibility coefficients of organic matter (OM), CP and P. All samples were kept at −20 °C until analysis. After thawing, the samples were weighed and dried in a forced air stove at 60 °C for 72 h. The DM of the samples was determined by the weight difference between the fresh matter and the DM. After drying, the samples were mill-ground and stored in watertight plastic bags until analysis.

### 2.5. Calculation of Apparent Digestibility, Estimation of Fecal Nitrogen Volatilization and Estimation of Urine Urea Nitrogen

The total digestive tract apparent digestibility of CP, OM and P was calculated using the nutrient-to-marker ratio in the diet and feces, as follows:

Apparent digestibility coefficient (%) = 100 − [100 × (Marker_diet_/Marker_feces_) × (Z_feces_/Z_diet_)]

where Z_feces_ and Z_diet_ are the nutrient concentrations (%) in the feces and in the diet, respectively. The Marker_feces_ and Marker_diet_ are the concentrations (%) of acid insoluble ash (AIA) in the feces and in the diet, respectively. The Z_diet_ and Marker_diet_ were calculated by considering the amount of concentrate and straw consumed per pen.

The apparent volatilization of fecal nitrogen (N) was estimated from the change in the N:P proportions of diet and feces following the equation used by Todd et al. [13]:

N volatilization (% of intake) = (N:P diet−N:P feces)/(N:P diet)

Urea nitrogen excreted in urine was estimated from blood urea concentrations according to the equation described in Institut National de la Recherche Agronomique (INRA) [7]:

Urine ureic nitrogen (g N/d/kg of BW) = (0.078 + 1.06 * urea N)/100

where urea N (mg/mL) is the nitrogenous fraction of plasma urea concentrations.

### 2.6. Chemical Analysis of Food and Feces

Feed and fecal DM (index no. 934.01), ash (index no. 942.05), ethereal extract (index no. 920.39) and starch (index no. 996.11) contents were determined according to AOAC methods [14]. NDF and ADF were analyzed with an Ankom200/220 [15] following the sequential procedure described by Van Soest et al. [16]. The NDF of the concentrates was analyzed, including a stable amylase at high temperatures (aNDF), and all values of NDF and ADF were corrected by discounting their ash content. The CP content of the feed (N × 6.25) was determined following the DUMAS procedure, using a nitrogen and protein analyzer (Model NA 2100, CE Instruments, Thermoquest SA, Barcelona, Spain). Phosphorus was determined by ultraviolet-visible spectroscopy (ICP-OES, HORIBA Jobin Yvon, Activa family, with AS-500 Autosampler, HORIBA Scientific, Madrid, Spain). Apparent digestibility of nutrients was estimated by AIA technique, which was determined following the procedure described in Álvarez-Rodríguez et al. [17]. All samples were analyzed in duplicate.

### 2.7. Histology and RT-qPCR Analysis

Immediately after slaughter, ruminal and ileal tissues were collected for histology and quantitative real-time PCR (qPCR) analysis from four lambs from CP20/19 group and four lambs from CP18/17 group. For histological examination, 5-cm^2^ samples from the cranial dorsal sac were taken, rinsed with phosphate-buffered saline solution (PBS) and fixed in a 10% formalin solution. For cytokine gene expression, several portions of ruminal tissue from the cranial dorsal sac and a 2-cm segment of the ileum proximal to ileocecal valve were divided, rinsed with PBS, incubated in RNAlater (Invitrogen, Madrid, Spain) and stored at −80 °C.

#### 2.7.1. Histological and Morphometric Analysis

Formalin-fixed tissue samples were trimmed and processed according to standard histological procedures, and sections were stained with hematoxylin-eosin. From each sample, two sections with 5 or more papillae were examined. Slides were examined with a Motic BA310E microscope and digital pictures were taken at 40× magnification with a digital camera (Moticam 1080) to determine the variations of the epithelial keratinization degree [18]. Thickness of epithelium and keratin layer was measured at 10 different sites in each picture using the image processing and analysis software (Motic Images Plus 3.0 ML, Kowloon, Hong Kong). Epithelial keratinization degree was calculated as the proportion that represented the keratin layer respecting the thickness of epithelium.

#### 2.7.2. Cytokine Gene Expression

Total RNA was extracted from 100 mg of ruminal and ileal tissues according to the method of Chomczynski and Sacchi [19]. Concentrations of RNA were determined spectrophotometrically. Samples were treated with DNAse in the presence of RNAse inhibitors to eliminate contaminating genomic DNA. Complementary DNA was synthesized from 1 µg of total RNA in the presence of random primers using the RevertAid H Minus First Strand cDNA synthesis Kit (Thermo Scientific, Waltham, MA, USA) according to the manufacturer’s recommendations.

Messenger RNA expression was determined by qPCR for three target genes—interleukin 10 (*IL10*), tumor necrosis factor-α (*TNFA*) and transforming growth factor-β1 (*TGFB*). The genes glyceraldehyde-3-phosphate dehydrogenase (*GAPDH*) and β-actin (*ACTB*) were used as housekeeping genes. Sequences of primers for *GAPDH* and *ACTB* have been described elsewhere [20]. Primers for *IL10*, *TNFA*, *TGFB* were designed with the Primer3Plus tool [21] and synthesized by Eurofins Genomics (Eurofins Genomics, Ebersberg, Germany) (Table 2). To avoid genomic contamination, all primers were designed to span an intron. For each gene, a standard curve was generated by amplifying serial dilutions of a control cDNA to check for linearity between initial template concentration and cycle threshold (Ct) values. Amplification was conducted using the SYBR green method of the ABI PRISM 7500 sequence detector (Applied Biosystem, Foster City, CA, USA) under the conditions specified by the manufacturer: an initial activation and denaturation step of 10 min at 95 °C followed by 40 cycles consisting of 10 s at 95 °C and 1 min at 60 °C. PCR reactions were run using 3 μL of 30-fold diluted cDNA as template in a total volume of 8 μL containing 1 × Maxima SYBR Green/ROX qPCR Master Mix (Thermo Scientific, Waltham, MA, USA) as reported elsewhere [22]. Each measurement was carried out in triplicate and the average used to calculate the relative gene amount. Data were normalized and analyzed by the 2^−ΔΔCt^ method using the mean Ct value obtained for the two reference genes and the Ct values for each cytokine primer [23]. The relative expression value was set to 1 for the CP20/19 group.

### 2.8. Statistical Analysis

For statistical analysis, individual data from 3 lambs in the CP18/17 group (one from batch 1 and two from batch 2) were removed due to death or pathological growth retardation. The data were analyzed with the statistical package JMP Pro13 (SAS Institute Inc. Cary, NC, USA). The growth and carcass performance, blood metabolites and nutrient digestibility data of individual animals were analyzed through mixed models with repeated measurements that included the treatment and the block (batch) as fixed effects, and the pen as a random effect. The feed intake, FCR and within-pen coefficient of variation of BW data of pens were analyzed with simple least squares models that included the same fixed effects as the mixed models. In all the evaluated variables, the pen was considered as the replicate (n = 12). The single interaction between the two fixed effects did not affect any parameter and was removed from the final model. The Student’s t-test or one-way ANOVA test were used to compare relative cytokine gene expression and epithelial keratinization degree for fixed effects and their interactions. Least square means and their standard error are described. The separation of means was carried out with Tukey′s test. The level of significance was set at 0.05, but tendencies were commented on if the level of significance was below 0.10.

## 3. Results

The effects of experimental diets on the productive performance of lambs is shown in Table 3, Table 4 and Table 5. The initial and final BW of the growing and finishing phases, as well as the ADG, were similar for both phases and experimental groups (Table 3; *p* > 0.05).

No significant differences were observed between the CP20/19 and CP18/17 groups in the average daily concentrate and straw intake during the growing and finishing phases (Table 4; *p* > 0.05).

The different levels of CP used in the CP20/19 and CP18/17 groups did not affect the FCR of the growing phase (Table 4; *p* > 0.05). However, the FCR of the finishing phase showed a tendency to be higher in the CP20/19 group compared to the CP18/17 group (Table 4). The overall FCR for the CP20/19 and CP18/17 treatments were 3.34 and 3.17 g/g ± 0.09 (*p* = 0.21), respectively.

The dietary CP level of the experimental diets did not affect the carcass weight and carcass dressing (Table 5; *p* > 0.05).

Blood urea concentration was higher in the CP20/19 group than in the CP18/17 group in both growing and finishing phases (Table 6; *p* < 0.05). Blood creatinine did not differ between groups in the growing phase, but it tended to be higher in the CP18/17 group of the finishing phase than in the CP20/19 group (*p* = 0.07). The U/C ratio was higher in the CP20/19 group than in the CP18/17, both in growing and finishing phases (*p* < 0.01). The plasma concentrations of free MDA, bound to proteins and total MDA, as biomarker aldehyde reflecting OS, were similar between the experimental diets in both feeding phases (Table 6; *p* > 0.05).

The dietary AIA content was 0.86 ± 0.05% and 0.80 ± 0.07% for CP20/19 and CP18/17 groups, respectively, that, in both cases, consumed a 13:87 forage to concentrate ratio. The percentage of DM in feces, as well as the apparent digestibility of CP in the growing and finishing phases did not differ between the CP20/19 and CP18/17 groups (Table 7; *p* > 0.05). The apparent digestibility of OM was lower in the CP20/19 than in the CP18/17 group during the growing and finishing phases (*p* < 0.05). On the other hand, the digestibility of P in the growing phase was not different between the CP20/19 and CP18/17 groups (*p* > 0.05). However, during the finishing phase, the apparent digestibility of P was lower in the CP20/19 than in the CP18/17 group (Table 7; *p* = 0.02).

The dietary N:P ratio showed differences between the CP20/19 and CP18/17 groups in both growing and finishing phases (Table 7; *p* < 0.05). Conversely, the reduction in dietary CP did not affect the N:P ratio of feces in any of the phases studied (*p* > 0.05). The estimated fecal volatilization of N tended to be higher in the growing phase (*p* = 0.07), and it was indeed significantly higher in the finishing phase (*p* < 0.05) in the CP20/19 compared to the CP18/17 group (Table 7). During the growing phase, the estimated ureic N in urine was different between the CP20/19 and CP18/17 groups (Table 7; *p* < 0.05). During the finishing phase, the estimated ureic N in urine tended to be higher in the CP20/19 than in the CP18/17 group (Table 7; *p* = 0.05).

The reduction of dietary CP did not affect the rumen epithelial keratinization degree (32.0 vs. 33.8 ± 2.9%, in CP20/19 and CP18/17 groups, respectively, *p* > 0.05).

**Figure 1:**
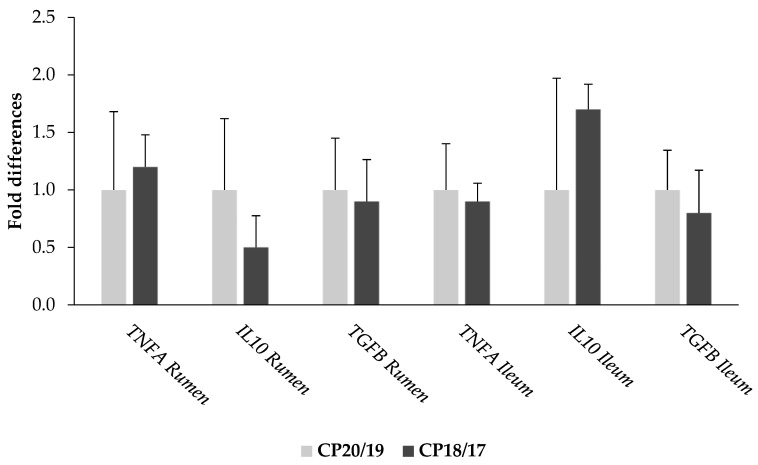
Relative fold differences in *TNFA*, *IL10* and *TGFB* gene expression according to the reduction of dietary CP. The CP20/19 group was designated as calibrator to compare expression levels. Bars represent least square mean values ± SE (CP20/19 vs. CP18/17; *p* > 0.05).

No effects of reduction of dietary CP were observed on cytokine expression (Figure 1). The CP18/17 group showed similar mRNA expression of *TNFA, IL10* and *TGFB* in ileal and ruminal tissues, as compared to that of the CP20/19 group (*p* > 0.05).

## 4. Discussion

This work was designed to assess the effects of tailoring dietary CP supply to the requirements of intensively-fed light lambs from a Spanish local sheep breed (Ripollesa), that are generally early maturing and small frame in size [24]. Therefore, the growth performance of this breed did not respond to the nutrient supply in the same manner as selected breeds whose protein requirements have been fitted through prediction equation models in several rationing systems (for instance, INRA or National Research Council).

Lambs fed CP18/17 CP had a similar ADG and final BW compared to lambs fed the CP20/19 diet, which had 2% greater dietary CP (20% vs. 18% CP and 19% vs. 17% CP on a DM basis, in growing and finishing phases, respectively). The concentrate and straw intake were not affected by dietary CP either. Similar results were obtained by Purroy et al. [25] in light lambs (15 to 25 kg of BW) from a close local sheep breed (Rasa Aragonesa) fed 15% and 18% dietary CP on a DM basis. However, they observed that feed intake was reduced when dietary CP decreased to 12% on DM basis. Low dietary CP strategies (up to 11% CP on DM basis) have been also used in heavy lamb production (<44 kg of final BW) without detrimental effects on growth performance compared to standard feed (16% dietary CP on a DM basis) [26], thereby improving farm profitability.

Despite no overall differences being detected, during the finishing phase there was a tendency for greater FCR in the CP20/19 group than in the CP18/17 group. This may be related to a disruption in nitrogen-energy balance in the rumen that could have impaired microbial protein synthesis [27], and consequently, the greater dietary CP in the CP20/19 group was not counterbalanced with an increased growth performance. The last INRA [7] feeding system for ruminants estimates that growing-finishing lambs from 15 to 25 of BW, with 250 g of ADG, requires between 110 and 103 g metabolizable protein PDI (protein digestible in the small intestine)/kg DM intake. This represents a dietary CP supply between 16.2% and 15.1% on a DM basis, respectively [7]. Recently, it has been observed that selected Romane lambs (with ADG > 320 g/day) can respond to increases up to 20% of dietary CP (on a DM basis) by improving their apparent CP digestibility and growth performance, although their FCR is similar to lambs fed 15% CP [28]. Thus, our findings suggest that FCR remains steady when the dietary CP is kept within the range of 13% to 18% on a DM basis, in agreement with studies performed in other breeds [25,28,29].

The dietary CP requirements may be also affected by the dietary energy content. In order to seize maximal growth potential from improved Merino crossbreeds, some authors suggest increasing the protein/energy ratio up to 140 g PDI/net energy supply value (UFV) [30]. However, the current Spanish nutritional guidelines for finishing lambs recommend dietary supplies ranging from 105 to 125 g PDI/UFV for light lambs and improved crossbred lambs, respectively [5]. In this study, this ratio ranged from 138 to 130 g PDI/UFV for the CP20/19 group and from 127 to 120 g PDI/UFV in the CP18/17 group, during growing and finishing phases, respectively. Indeed, the response to nutrient supply would be dependent on the genetic growth potential and the health status of the lambs. Therefore, it is not expected that the current autochthonous breeds raised in Spain respond to a much greater dietary CP supply.

Neither the BW at slaughter nor carcass dressing proportion differed between dietary CP groups. These results are in agreement with studies performed in heavy lambs (32 to 44 kg of BW) that were fed 11% and 16% CP diets (on DM basis) [26].

Blood urea is considered to be an indicator of ingested (dietary) or mobilized (body) protein, while circulating creatinine highlights the creatine degradation which is involved in muscle energy metabolism [31]. During the growing and finishing phases, the circulating urea concentrations were greater in the CP20/19 group compared to the CP18/17 group. Blood urea is a hepatic by-product of protein breakdown resulting from dietary CP supply and body protein utilization [32]. This explains the greater circulating urea in the CP20/19 group compared to the CP18/17 group lambs. However, circulating creatinine, which may be a skeletal muscle biomarker [33], did not differ between treatments during the growing phase but tended to be lower in the CP20/19 group than in the CP18/17 group. This may suggest certain differences between the groups in daily muscle turnover. The observed blood urea concentration was in line with Mahmoud [34], who concluded that lambs fed 17% dietary CP showed greater blood urea than those receiving 14% and 11% dietary CP (on DM basis). A reduction of dietary CP also triggers lower ruminal NH_3_-N concentration and CH_4_ production [35].

The dietary CP reduction did not affect fecal consistency, as the dry matter content of feces was similar across groups. However, the apparent digestibility of organic matter was lower in the CP20/19 group than in the CP18/17 group, both in growing and finishing phases. Previous studies evidenced a curvilinear relationship between dietary CP and the apparent digestibility of OM, with maximum outcomes at 18% CP (on a DM basis) but lower values above and below this point [36,37]. The apparent digestibility of CP was not affected by dietary CP reduction during the growing and finishing phases. According to Haddad et al. [36], a decrease in the apparent digestibility of CP was observed when dietary CP was set at 10%.

Excessive dietary CP increases environmental load by ruminants [13], since between 30% and 50% of N intake may be affected by volatilization, especially through NH_3_ loss [38]. The estimated fecal N volatilization tended to be higher in the CP20/19 group than in their CP18/17 counterparts. Similar results were obtained when using the same predictive equation of Cole et al. [38] in beef steers fed different dietary CP levels (11.5 vs. 13% of CP). Therefore, nitrogen supply, as some other minerals as P, may be matched to requirements to reduce their excretion in manure [3]. In this study, the dietary N:P ratio was greater in the CP20/19 group than in the CP18/17 group both in growing and finishing phases. However, the differences in the estimated fecal N volatilization were more marked in the finishing than in the growing phase.

Blood urea nitrogen may be used to estimate the excreted urinary nitrogen, which may be maintained between 0.20 and 0.30 g N/day/kg of BW to meet protein requirements and reduce nitrogen excretion [7]. In this study, the estimated urinary nitrogen excreted based on INRA [7] was 0.28 and 0.23 g N/day/kg of BW for CP20/19 group, and 0.23 and 0.21 g N/day/kg of BW for CP18/17 group, in growing and finishing phases, respectively. This calculation suggests that lambs fed CP18/17 CP were more efficient in dietary CP use. The reduction of dietary CP seems a feasible strategy to mitigate the nitrogen gas emissions, as both NH_3_ (an environmental acidifier) and N_2_O (a potent warming gas aroused from the NH_4_ nitrification–denitrification process) may be reduced [27].

Characterization of blood OS is required to understand nutrient metabolism when nutrition is challenged and how this may influence the growth performance. In this regard, the blood OS may be considered a metabolic marker in animal health [39]. Severe protein restriction has been associated with oxidative stress status [8], leading to the production of free radicals and pro-inflammatory cytokines, which may damage the ruminal and intestinal tissue. Thus, it is expected that blood MDA, a lipid oxidation end product, increases in OS condition [11]. Nevertheless, in this study, there were no differences in this blood compound between groups in any phase. This may suggest that the evaluated dietary CP reduction did not trigger either a blood oxidative stress or dysfunctional pro-inflammatory responses in the gastrointestinal tract. Immune function in ileal and ruminal tissues was not compromised and no differences were observed in proinflammatory and anti-inflammatory cytokine expression as a consequence of a 2% protein reduction. On the other hand, increased parakeratosis has been also observed in lambs in response to diets with concentrate (19.9% CP on DM) vs. grazing alfalfa (24.7% CP on DM) [40]. However, as noted for keratin layer in ruminal epithelium, ruminal histomorphogenesis remained unaltered in this study. Hence, management practices related to improving protein content in light lamb diets seemed here not to affect the interplay between nutrition, metabolism and immune function.

## 5. Conclusions

In conclusion, a 2% reduction of dietary CP in intensively-raised light lambs did not impair their growth performance, reduced blood urea without affecting their oxidative status or proinflammatory and anti-inflammatory cytokine gene expression, and improved the apparent digestibility of organic matter and phosphorus.

## Figures and Tables

**Table 1:** Ingredients and Chemical Composition of the Experimental Diets.

	Growing (14–19 kg of BW)		Finishing (19–25 kg of BW)	
	20% CP	18% CP	19% CP	17% CP
**Ingredients**				
Wheat	29.9	30.0	29.9	29.9
Barley	20.5	21.8	23.1	25.5
Maize	20.5	21.9	23.3	23.6
Soybean meal 47	16.0	13.3	10.7	7.9
Dry maize distillery grains	6.0	6.0	6.0	6.0
European rapeseed meal	3.0	3.0	3.0	3.0
Calcium carbonate	2.3	2.3	2.4	2.4
Salt	0.5	0.5	0.5	0.5
Ammonium chloride	0.5	0.5	0.5	0.5
Vitamin-mineral premix *	0.3	0.3	0.3	0.3
Oil/surfactant premix	0.2	0.2	0.2	0.2
**Analyzed Chemical Composition**				
Dry matter (DM, % on fresh matter)	88.6	89.2	90.9	90.4
Ash (% on DM basis)	5.6	5.9	5.4	5.5
Crude protein (% on DM basis)	20.4	18.3	19.1	17.4
Ether extract (% on DM basis)	2.87	2.67	2.81	2.69
Starch (% on DM basis)	49.2	49.9	52.2	55.9
Neutral-detergent fiber (% on DM basis)	14.5	13.6	14.2	13.8
Acid-detergent fiber (% on DM basis)	5.3	4.8	4.6	4.6
Phosphorus (% on DM basis)	0.42	0.45	0.42	0.42

* Ingredients of vitamin-mineral premix contained in each kilogram of feed: Vitamin A, 9900 UI; Vitamin D_3_, 2250 UI; Vitamin E, 300 UI; Vitamin B1, 1 mg; Zn, 77.7 mg; Mn, 35.6 mg; Cu, 2.2 mg; Co, 0.96 mg; I, 0.62 mg; Se, 0.20 mg; BHT (E321), 62.5 mg and Propyl gallate (E310), 7.5 mg.

**Table 2:** Primer Sequences for *IL10*, *TGFB*, *TNFA*, *GAPDH* and *ACTB* Used for Quantitative Real-Time PCR.

Gene	Forward and Reverse Primer (5′–3′)	bp	Access. No.	E (%)	n M	Source
*GAPDH*	F: ATCTCGCTCCTGGAAGATG R: TCGGAGTGAACGGATTCG	200	NM_001190390.1	1.90	600 300	[20]
*ACTB*	F: CTGGACTTCGAGCAGGAGAT R: GATGTCGACGTCACACTTC	194	NM_001009784	1.94	600	[20]
*IL10*	F: TTAAGGGTTACCTGGGTTGC R: TTCACGTGCTCCTTGATGTC	109	NM_001009327.1	1.96	200	[21]
*TGFB*	F: TTGACGTCACTGGAGTTGTG R: CGTTGATGTCCACTTGAAGC	120	NM_001009400.2	2.04	200	[21]
*TNFA*	F: CAAATAACAAGCCGGTAGCC R: TGGTTGTCTTTCAGCTCCAC	118	NM_001024860.1	1.96	200	[21]

Note: F, Forward; R, Reverse; bp, amplified product length in base pairs; E (%), Efficiency; nM, Optimal primer concentration

**Table 3:** Effect of Experimental Diets on Body Weight (BW) and Average Daily Gain (ADG).

Item	CP20/19	CP18/17	SE	*p*-Value
**Growing (14 to 19 kg)**				
Initial BW (kg)	15.0	15.0	0.15	0.97
Within-pen coefficient of variation of BW (%)	5.8	6.5	0.73	0.49
ADG (g)	235	234	13.0	0.92
**Finishing (19 to 25 kg**)				
Initial BW (kg)	19.8	19.7	0.28	0.75
Within-pen coefficient of variation of BW (%)	9.6	10.0	1.09	0.83
ADG (g)	254	269	8.3	0.23
Slaughter BW (kg)	24.5	24.8	0.24	0.40
Within-pen coefficient of variation of BW (%)	8.6	8.0	0.85	0.60

Note: CP20/19 group was supplied 20% of dietary CP on DM and 19% of CP on DM in growing and finishing phases, respectively, whereas CP18/17 group was supplied 18% of dietary CP on DM and 17% of CP on DM in growing and finishing phases, respectively. SE = Standard error.

**Table 4:** Effect of Experimental Diets on Average Daily Concentrate and Straw Intake and Feed Conversion Rate (FCR).

Item	CP20/19	CP18/17	SE	*p*-Value
**Growing (14 to 19 kg)**				
Concentrate intake (g/day)	715	710	22	0.89
Straw intake (g/day)	108	107	6	0.97
FCR (g/g)	3.11	3.06	0.13	0.82
**Finishing (19 to 25 kg)**				
Concentrate intake (g/day)	878	854	24	0.48
Straw intake (g/day)	125	125	8	0.95
FCR (g/g)	3.50^x^	3.21^y^	0.11	0.07

Note: CP20/19 group was supplied 20% of dietary CP on DM and 19% of CP on DM in growing and finishing phases, respectively, whereas CP18/17 group was supplied 18% of dietary CP on DM and 17% of CP on DM in growing and finishing phases, respectively. The FCR was calculated as the ratio between average daily concentrate intake and ADG. ^x, y^ = *p* < 0.10; SE = Standard error.

**Table 5:** Effect of Experimental Diets on Slaughter Weight, Carcass Weight and Carcass Dressing.

Item	CP20/19	CP18/17	SE	*p*-Value
Slaughter weight (kg)	24.8	24.9	0.27	0.41
Carcass weight (kg)	11.8	11.7	0.19	0.43
Carcass dressing (%)	47.8	47.0	0.69	0.47

Note: CP20/19 group was supplied 20% of dietary CP on DM and 19% of CP on DM in growing and finishing phases, respectively, whereas CP18/17 group was supplied 18% of dietary CP on DM and 17% of CP on DM in growing and finishing phases, respectively. These carcass variables take into account half of the experiment animals (60 out of 120) that were allotted in 24 pens (12 replicates per dietary treatment). SE = Standard error.

**Table 6:** Effect of Experimental Diets on Metabolites Related to the Protein Nutritional Status and on the Oxidative Status of the Lambs.

Item	CP20/19	CP18/17	SE	*p*-Value
**Growing (14 to 19 kg)**				
Urea (mg/dL)	41 ^a^	32.3 ^b^	1.19	0.0004
Creatinine (mg/dL)	0.84	0.84	0.02	0.93
Rate U/C	48.8 ^a^	38.8 ^b^	1.59	0.0012
FMDA (µM/L)	0.52	0.54	0.01	0.27
PBMDA (µM/L)	6.77	6.85	0.21	0.78
TMDA (µM/L)	7.30	7.40	0.21	0.73
**Finishing (19 to 25 kg)**				
Urea (mg/dL)	32.5 ^a^	28 ^b^	1.45	0.05
Creatinine (mg/dL)	0.79 ^x^	0.88 ^y^	0.03	0.07
Rate U/C	40.4 ^a^	29.5 ^b^	1.56	0.0023
FMDA (µM/L)	0.66	0.68	0.05	0.85
PBMDA (µM/L)	6.39	6.74	0.31	0.44
TMDA (µM/L)	7.06	7.43	0.32	0.43

Note: CP20/19 group was supplied 20% of dietary CP on DM and 19% of CP on DM in growing and finishing phases, respectively, whereas CP18/17 group was supplied 18% of dietary CP on DM and 17% of CP on DM in growing and finishing phases, respectively. FMDA = Free Malondialdehyde; PBMDA = Protein-Bound Malondialdehyde; TMDA = Total Malondialdehyde; ^a, b^ = *p* < 0.05; ^x, y^ = *p* < 0.10; SE = Standard error.

**Table 7:** Effect of Experimental Diets on Dry Matter (DM) of Feces, Apparent Digestibility of Organic Matter (OM), Crude Protein (CP) and Phosphorus (P), Nitrogen-Phosphorus Rate (N:P) of Diet, N:P of Feces, Volatilization of Nitrogen (N) and Estimated Ureic N.

Item	CP20/19	CP18/17	SE	*p*-Value
**Growing (14 to 19 kg)**				
DM of feces (DM; %)	32.9	32.7	0.72	0.91
Digestibility of OM (%)	67.7 ^a^	71.4 ^b^	1.21	0.04
Digestibility of CP (%)	59.1	61.4	2.15	0.46
Digestibility of P (%)	37.7	39.5	4.37	0.77
N:P of diet	7.89 ^a^	6.85 ^b^	0.003	0.0001
N:P of feces	4.03	4.11	0.25	0.83
Fecal N volatilization (% of intake)	48.8 ^x^	39.9 ^y^	3.30	0.07
Estimated ureic N (g/N/d/kg)	0.28 ^a^	0.24 ^b^	0.005	0.0004
**Finishing (19 to 25 kg)**				
DM of feces (%)	32.6	33.3	0.91	0.58
Digestibility of OM (%)	64.1 ^a^	69.4 ^b^	1.64	0.03
Digestibility of CP (%)	55.1	60.1	2.43	0.17
Digestibility of P (%)	21.2 ^a^	37.9 ^b^	4.71	0.02
N:P of diet	7.14 ^a^	6.84 ^b^	0.002	0.0001
N:P of feces	3.69	4.18	0.22	0.13
Fecal N volatilization (% of intake)	48.2 ^a^	38.8 ^b^	3.21	0.049
Estimated ureic N (g/N/d/kg)	0.24 ^x^	0.22 ^y^	0.007	0.050

Note: CP20/19 group was supplied 20% of dietary CP on DM and 19% of CP on DM in growing and finishing phases, respectively, whereas CP18/17 group was supplied 18% of dietary CP on DM and 17% of CP on DM in growing and finishing phases, respectively. ^a, b^ = *p* < 0.05; ^x, y^ = *p* < 0.10; SE = Standard error.
